# Sibling, Peer, and Cyber Bullying Among Children and Adolescents: Co-occurrence and Implications for Their Adjustment

**DOI:** 10.3389/fpsyg.2021.761276

**Published:** 2021-12-22

**Authors:** Slava Dantchev, Martina Zemp

**Affiliations:** Department of Clinical and Health Psychology, University of Vienna, Vienna, Austria

**Keywords:** bullying, siblings, peer, cyber, well-being, academic achievement

## Abstract

Bullying across the sibling, peer, and cyber context has consistently been associated with a range of long-term health and well-being consequences for children and adolescents. Although research examining different bullying forms simultaneously in the same study are emerging, it remains unclear to what extend sibling, peer, and cyber bullying co-occur and in what ways they are associated. Moreover, previous work has demonstrated that children and adolescents who experience multiple forms of victimization are at a particular risk of adverse outcomes. However, whether different constellations of co-occurring bullying forms have differential impacts has not yet been investigated sufficiently. The aim of the present study was to examine the frequencies of isolated and co-occurring sibling, peer, and cyber bullying as well as to explore their independent and cumulative relationships with child adjustment. This study was based on a sample of 329 children and adolescents aged between 9 and 15. Bullying experiences across the sibling, peer, and cyber context in the previous 6 months were assessed *via* self-report. Youth further reported on emotional problems, conduct problems, sleep problems, and academic achievement *via* an online questionnaire. Sibling, peer, and cyber bullying were uniquely associated with child outcomes. A cumulative relationship between bullying victimization across contexts and emotional problems, conduct problems, and sleep problems could be identified, while bullying perpetration across contexts was only linked to more conduct problems in a cumulative manner. The findings have important practical implications arguing for the adoption of a holistic approach toward bullying in prevention and intervention.

## Introduction

According to Olweus (1996), bullying is defined as aggressive behavior or intentional harm doing which is carried out repeatedly over time in an interpersonal relationship characterized by an imbalance of power. Bullying has been documented between siblings in the home (Wolke et al., 2015), between peers in school (Wolke and Lereya, 2015), and *via* electronic media, also known as cyber bullying (Olweus, 2012). Bullying between siblings and peers can take on direct (e.g., hitting, name calling or teasing) or indirect (e.g., spreading rumors or social exclusion) forms, while cyber bullying makes use of electronic means (e.g., text messages or social media). Youth may be involved in bullying in different ways; they may experience victimization (i.e., those who are bullied), become involved in perpetration (i.e., those who bully others), or both behaviors together (i.e., a specific sub-group of children referred to as bully-victims).

Prevalence rates range from 15 to 50% for sibling victimization and 10 to 40% for sibling perpetration (Wolke et al., 2015). In the peer context a mean prevalence rate of 35% has been reported for any involvement, whereas 15% has been reported for cyber bullying involvement according to meta-analytic data (Modecki et al., 2014), with prevalence estimates for victimization typically being higher compared to perpetration in both contexts (Jadambaa et al., 2019; Lebrun-Harris et al., 2019). Representative population studies agree, however, that victimization by siblings is reported more frequently than by peers (Finkelhor et al., 2015; Dantchev et al., 2019). Previous studies have consistently reported a homotypic relationship (i.e., perpetrating aggression toward a sibling is linked to perpetrating aggression toward a peer, for instance) between sibling and peer bullying (Tippett and Wolke, 2015; Dantchev et al., 2019). Emerging longitudinal data suggest that sibling victimization in early childhood precedes peer victimization in middle childhood and adolescence (Tucker et al., 2019). Similarly, peer and cyber bullying have also been found to overlap strongly (Modecki et al., 2014). Consequently, some scholars argue that cyber bullying should be understood as an extension of traditional peer bullying (Wolke et al., 2017). Whether and how sibling and cyber bullying are associated is still unknown. In order to make more accurate comparisons and gain a better understanding of the prevalence and interrelation between sibling, peer, and cyber bullying, studies that measure these constructs together within the same sample are needed.

Bullying is considered a major public health issue that is associated with long-term economic costs and consequences for society (Brimblecombe et al., 2018; Jadambaa et al., 2019). A large body of research underlines the harmful outcomes related to bullying experiences in childhood (Moore et al., 2017). Moreover, there is robust evidence supported by meta-analytic data showing that bullying victimization and perpetration is a predictor of mental disorders (McKay et al., 2021), sleep problems (van Geel et al., 2016; Zimmer-Gembeck et al., 2019), and criminal or delinquent behavior (Ttofi et al., 2012). Bullying victimization has furthermore been identified as a predictor of poorer academic achievement (Nakamoto and Schwartz, 2010; Kowalski et al., 2014). The consensus is, so far, that youth experiencing bullying victimization are more at risk of internalizing disorders, while those who perpetrate bullying are at increased risk of externalizing behaviors (Klomek et al., 2015). It is important to consider, however, that the large majority of studies have referred to peer bullying exclusively, which makes it difficult to generalize the findings to the sibling and cyber context. More research is necessary to explore the links between sibling and cyber bullying and child adjustment to fill this gap, especially in the domains of sleep problems and academic outcomes.

There is a breadth of proposed mechanisms that have been put forward in an effort to explain why bullying may be associated with poor child adjustment. Firstly, there is a growing body of empirical work demonstrating that bullying should be considered as an additional form of childhood trauma (Idsoe et al., 2021; McKay et al., 2021), similarly contributing toward cognitive and physiological changes that are directly linked toward the development of poor physical and mental health (Arseneault, 2017). On par with this, sleep problems may be understood as both a potential consequence of bullying involvement, but also as a causative factor that can contribute to the occurrence of bullying (Donoghue and Meltzer, 2018). Along these lines, bullying has been argued to function as a significant stressor for youth. Worry and rumination of future victimization may for example directly influence child and adolescent sleep quality (Kubiszewski et al., 2014). At the same time poor sleep quality in childhood and adolescence has been linked to problems with emotional and behavioral regulation and aggression (Dahl and Lewin, 2002; Krizan and Herlache, 2015). Moreover, from a preventative standpoint it is essential to consider the wider social environment of children and adolescents and the ways in which they relate and interact with one another at home, in school or online. In line with social learning theory (Bandura, 1977), aggressive behavior that is reinforced may be modeled in other contexts, thus exploring the joint interplay between bullying behavior across contexts is pivotal.

Comparative studies examining the links between child adjustment and bullying across different contexts simultaneously are relatively scarce. Existing studies, however, suggest that bullying across the sibling and peer context are associated with similar negative outcomes for children and adolescents in relation to sub-clinical emotional and behavior problems, as well as mental health problems including anxiety, depression, suicidal ideation, and self-harm (Tucker et al., 2014; Dantchev et al., 2018; Dantchev and Wolke, 2019; Dantchev et al., 2019; Foody et al., 2020). Contrary to this, findings on bullying across the peer and cyber context are inconsistent. Some studies have reported a similar influence of peer and cyber bullying on emotional and behavioral problems (Wolke et al., 2017), others have found differential effects (Kowalski and Limber, 2013), and some have found a weaker link for cyber bullying (Kubiszewski et al., 2015). Yet others have suggested that cyber bullying does not have a negative effect on child outcomes over and above peer bullying (Olweus, 2012). To our knowledge, there are no studies thus far that have examined all three bullying contexts (namely sibling, peer, and cyber) within the same sample and investigated their independent relationship with child outcomes whilst controlling for one another. This is crucial, however, as such data could reveal whether specific bullying contexts contribute uniquely toward child adjustment.

An additional caveat of the current literature is that there are no previous studies that have explored the cumulative effects between all three bullying forms and child adjustment. Previous work has shown that children who are victimized by their siblings and peers are at a greater risk of developing poor mental health and suicidality (Tucker et al., 2014; Dantchev et al., 2018; Dantchev and Wolke, 2019; Dantchev et al., 2019; Foody et al., 2020; Sharpe et al., 2021). Those who perpetrate bullying both at home and at school are more likely to display criminal behavior and engage in illicit drug use (Dantchev and Wolke, 2019). Similarly, children and adolescents that are victimized concurrently by peers and in the cyber context have been reported to display the greatest emotional and behavioral problems (Wolke et al., 2017). Identifying a cumulative association between multiple forms of bullying across contexts have important practical implications. Successfully reducing bullying in one context may in turn have a positive carry-over effect toward reducing bullying in another context. There is evidence suggesting that if victimization ceases, children and adolescents may return to a similar mental health state as those who have never been victimized (Sharpe et al., 2021). Therefore, understanding how bullying across contexts are interlinked is key for the development of effective anti-bullying programs by taking on a multi-modal approach.

The aim of the present study is to provide novel data that incorporates sibling, peer, and cyber bullying from a sample of children and adolescents living in Austria in order to provide a holistic overview of multiple bullying forms. The following four research questions will be addressed: (1) How frequent do children and adolescents report isolated and co-occurring forms of bullying (victimization and perpetration)? We hypothesized that sibling bullying would be reported most frequently, followed by peer and then cyber bullying. We further expected youth to report co-occurring bullying more frequently compared to isolated bullying forms. (2) How are sibling, peer, and cyber bullying (victimization and perpetration) related? We expected that there would be a homotypic relationship between bullying across contexts (e.g., the more victimization in one context, the more victimization in another context). (3) Are sibling, peer, and cyber bullying (victimization and perpetration) associated with child adjustment? It was hypothesized that youth reporting any sibling, peer, or cyber bullying involvement would experience more emotional problems, conduct problems, sleep problems, as well as lower levels of academic achievement, even after accounting for one another. (4) Is there a cumulative relationship between bullying victimization and perpetration across the sibling, peer, and cyber context and child adjustment? Cumulative associations between exposure to multiple forms of bullying victimization and perpetration across contexts and child outcomes were expected.

## Materials and Methods

### Participants

Our starting sample consisted of all children who completed our questionnaire (*N* = 494). In order to participate in the study, children and adolescents had to be aged between 9 and 15 years and provide reports about their family constellation (e.g., whether they had a sibling), resulting in a sample of 395 youth (*M* = 12.52; *SD* = 1.29). Out of these children, 66 (16.7%) reported that they had no siblings. These children were excluded from all further analysis, because sibling bullying was one of the central variables of the focus of this report. The final sample used for this study thus consisted of 329 children and adolescents (50.6% male) who had at least one sibling (see Supplementary Figure 1 for participant flowchart). Around half of the participants reported having one sibling (52.9%), a third had two siblings (32.5%), 7.9% had three siblings and 6.5% had four or more siblings. Moreover, 38.9% of youth reported being the first-born child. Children and adolescents were asked to report with whom they currently lived in the same household (multiple selections were possible): The majority of children and adolescents reported living in a household with their biological mothers (96.7%), followed by their biological fathers (80.2%), siblings (85.1%), and finally stepparents (6.7%). Approximately two thirds (62.6%) of children and adolescents reported attending a grammar school, 33.7% reported attending a secondary modern school, and 3.6% reported attending another school form.

### Procedure

The current study employed an online survey design using the software SoSci Survey (Leiner, 2019). Two separate study links were created in order to allow independent participation of parents and their children, but only child-reports are used for this report. Participants were recruited *via* two routes: (1) collaborations with Austrian schools; (2) social media platforms for parents and families. Austrian schools were randomly selected and initially contacted *via* telephone in order to provide an overview of the study rationale. A total of 14 schools were willing to participate in circulating a standardized e-mail including a study cover letter, the study leaflet, and the link to the online survey to parents. Moreover, recruitment materials were shared on social media platforms. Two recruitment phases were carried out. The first phase took place between April 2020 and June 2020, while the second phase took place between January 2021 and April 2021. In order to ensure that parental consent of all participating youth was obtained, the study link for children and adolescents was only available to those parents who had read through the study rationale and agreed to share the study link with their children. Parents were thus given the choice of participating themselves and/or sharing the designated study link with their children. Ethical approval for the study was obtained from the Institutional Review Board of the University of Vienna (Reference Nr.: 00480; Date of approval: 19.11.2019).

### Measures

#### Sibling, Peer, and Cyber Bullying

Sibling bullying victimization and perpetration was assessed *via* two items that were adapted and translated into German from the Revised Olweus Bullying Questionnaire (Olweus, 1996). Single item scales and multi-item scales of the sibling bullying questionnaire have been found to correlate highly with this measure (e.g., within the Avon Longitudinal Study of Parents and Children: victimization: *r* = 0.91, *n* = 6,909, *p* < 0.001; perpetration: *r* = 0.85, *n* = 6,856, *p* < 0.001; Boyd et al., 2013; Fraser et al., 2013). Thus, there is evidence for the validity of this short scale as also seen through its application elsewhere (Dantchev et al., 2018; Toseeb et al., 2020). Youth were first told that sibling bullying is “when a brother or sister tries to upset [them] by saying nasty and hurtful things, or completely ignores [them] from their group of friends, hits, kicks, pushes or shoves [them] around, tells lies or makes up false rumors about [them]”. They were then asked to report how frequently they had experienced sibling bullying (victimization) and whether they had ever bullied a sibling (perpetration) in the past 6 months. Responses were given on a 4-point Likert-scale (1 = *never*; 2 = *seldom: 1–3 times during past 6 months*; 3 = *frequently: more than three times during past 6 months*; 4 = *very frequently: at least once per week*).

Peer and cyber bullying victimization and perpetration were assessed *via* brief German-language bullying screening (Dantchev and Wolke, 2019; Kranhold et al., 2021). The screening is a short adapted version of a validated bullying questionnaire (Wolke et al., 2001) that was developed in a large population study in the United Kingdom and Germany. The screening includes six items that ask about the frequency of (1) direct peer bullying; (2) indirect peer bullying, and (3) cyber bullying. Youth were asked to report how frequently they had experienced each form of bullying (victimization) and whether they had ever bullied a peer in such ways (perpetration) in the past 6 months. Responses were given on a 4-point Likert-scale (1 = *never*; 2 = *seldom: 1–3 times during past 6 months*; 3 = *frequently: more than three times or more during past 6 months*; 4 = *very frequently: at least once per week*). Definitions and examples were given for each bullying form. The items assessing direct peer bullying victimization and perpetration are provided as an illustrative example:

“The following questions are about direct bullying. This means that others are harmed through a direct attack. Some young people may experience the following things repeatedly at school:

• They have been threatened or blackmailed
• They have been called bad or nasty names
• They have had nasty tricks played on them
• They have been hit or beaten up

How many times have you experienced these things in the past 6 months?

How many times have you done these things in the past 6 months?”

Children’s involvement in bullying within each context (sibling, peer, and cyber) was coded as present if reported more than three times in the past 6 months, in accordance to pre-established cut-offs (Wolke et al., 2001). Thus, six dichotomous bullying variables were computed: (1) sibling victimization, (2) sibling perpetration, (3) peer victimization (if direct and/or indirect forms were reported frequently), (4) peer perpetration (if direct and/or indirect forms were reported frequently), (5) cyber victimization, and (6) cyber perpetration (coded as: 0 = three times or less in the past 6 months; 1 = more than three times in the past 6 months). In addition, a bullying victimization index was computed by summing up the three dichotomous victimization variables, resulting in a possible range from 0 to 3 (*M* = 0.29, *SD* = 0.57), with each unit increase corresponding to an additional bullying form. The same procedure was applied to compute a perpetration index (*M* = 0.13, *SD* = 0.40). The victimization index was not normally distributed, with skewness of 2.13 (*SE* = 0.13) and kurtosis of 4.80 (*SE* = 0.27). The perpetration index was not normally distributed either, with skewness of 3.41 (*SE* = 0.13) and kurtosis of 13.54 (*SE* = 0.27). Thus, both indices were positively skewed, suggesting that co-occurring bullying experiences were rare. It is not uncommon that composite scores or indices reflecting cumulative childhood trauma are not normally distributed, as the prevalence of these experiences is comparatively low in the general population (see Shevlin et al., 2008; Radford et al., 2013). Moreover, it is common practice for scholars to compute an index or composite in order to reflect cumulative bullying (Dantchev et al., 2018; Dantchev and Wolke, 2019; Dantchev et al., 2019) or other traumatic experiences in childhood (Shevlin et al., 2008; Radford et al., 2013). Utilizing strict and consistent cut-offs in order to delineate between occasional conflict and bullying is critical. Therefore, applying the cut-off “if reported more than three times in the past 6 months” is important, even when exploring cumulative effects. By constructing dichotomous variables, we are including only those children into our victimization index and perpetration index who reported frequent bullying experiences. Thus, the conclusion we draw from our results can be directly linked to bullying behavior per definition (e.g., each unit increase in the index corresponds to an additional bullying form experienced).

#### Emotional Problems

Emotional problems were assessed by self-report *via* the five-items subscale of the Strengths and Difficulties Questionnaire (SDQ; Goodman, 2001) adapted for use in German (Lohbeck et al., 2015). Children and adolescents were asked to think about their behavior over the last 6 months and indicate to what extent a list of statements applied to them. Emotional problems included: (1) “I get a lot of headaches, stomach-aches or sickness”; (2) “I worry a lot”; (3) “I am often unhappy, depressed, or tearful”; (4) “I am nervous in new situations. I easily lose confidence”; (5) “I have many fears, I am easily scared.” Responses were given on a 3-point Likert-scale with 1 = *not true*, 2 = *somewhat true*, and 3 = *certainly true*. A mean score was computed in order to reflect emotional problems, resulting in a possible range from 1 to 3, with higher scores representing greater emotional problems (*M* = 1.54, *SD* = 0.48). Cronbach’s alpha was 0.75 in the current sample.

#### Conduct Problems

Conduct problems were similarly assessed *via* the five-items subscale of the Strengths and Difficulties Questionnaire (SDQ; Goodman, 2001) according to youth self-reports. Conduct problems included: (1) “I get very angry and often lose my temper”; (2) “I usually do as I am told” (reverse coded); (3) “I fight a lot. I can make other people do what I want”; (4) “I am often accused of lying or cheating”; (5) “I take things that are not mine from home, school or elsewhere.” Responses were given on a 3-point Likert-scale with 1 = *not true*, 2 = *somewhat true*, and 3 = *certainly true.* In order to improve the internal consistency of this subscale, item 2 was removed and the subscale was computed with the remaining four items. A mean score was computed in order to reflect conduct problems, resulting in a possible range from 1 to 3, with higher scores representing greater conduct problems (*M* = 1.28, *SD* = 0.34). Cronbach’s alpha was 0.58 in the current sample.

#### Sleep Problems

Sleep problems were assessed *via* a subscale from the German-language Sleep Inventory for Children and Adolescents (SIKJ*;* Lehmkuhl et al., 2015). Children and adolescents were asked to think about their sleep behavior in the last 6 months and indicate to what extent a list of statements applied to them. Sleep problems included: (1) “I have problems falling asleep in the evening”; (2) “I have problems staying asleep during the night”; (3) “I feel restless in the night”; (4) “I wake up in the night and find it difficult to get back to sleep”; (5) “I have nightmares and remember them.” Responses were given on a 3-point Likert-scale with 1 = *not true*, 2 = *somewhat true/sometimes true*, and 3 = *certainly true/often true*. A mean score was computed in order to reflect sleep problems, resulting in a possible range from 1 to 3, with higher scores representing greater sleep problems (*M* = 1.47, *SD* = 0.49). Cronbach’s alpha was 0.80 in the current sample.

#### Academic Achievement

Academic achievement was assessed by asking children and adolescents to appraise their current academic achievement in relation to a list of school subjects: (1) German; (2) Mathematics; (3) Geography and Economics; (4) History and Sociology; (5) English. Responses were given on a 5-point Likert-scale in accordance to the Austrian educational system 1 = *excellent*; 2 = *good*; 3 = *satisfactory*; 4 = *sufficient*; 5 = *fail*. A mean score was computed in order to reflect subjective academic achievement, resulting in a possible range from 1 to 5, with higher scores representing lower academic achievement. Children were also asked to report their current school grades as a measure of objective academic achievement in the same way as described above. Cronbach’s alpha for both subjective (α = 0.87) and objective (α = 0.87) academic achievement were comparable. Moreover, subjective and objective measures were highly correlated (*r* = 0.87), thus the subjective measure (*M* = 3.89, *SD* = 0.80) was used for all analyses for the sake of simplicity.

#### Control Variables

Across all statistical models, youth sex (0 = female; 1 = male), age (in years), birth order (0 = later born, 1 = first born), number of siblings, and whether siblings lived in the same household (0 = no, 1 = yes) were included as control variables. Moreover, it is important to consider that the data in this study was collected during the COVID-19 pandemic and the majority of youth were affected by alternating periods of home schooling. School closures were incurred around mid-March 2020 in Austria and replaced by a hybrid solution around mid-May 2020. Hybrid solutions involved alternating periods during which half of the classroom was permitted to attend school while the other half attended digital home-schooling sessions. Youth were again permitted to attend school starting September 2020 under consideration of safety and health measures. National school closures were announced once more in November 2020. As of February 2021, another period of hybrid schooling was enforced. To account for these schooling irregularities as well as any pandemic-related influences we accounted for the recruitment phase (0 = April 2020 through June 2020; 1 = January 2021 through April 2021).

### Missing Data

A total of 93.9% of the sample had complete data on all variables across the entire survey. Missing data in the structural equation models were treated by applying the full information maximum likelihood (FIML) approach in Mplus.

### Statistical Analysis

**FIGURE 1:**
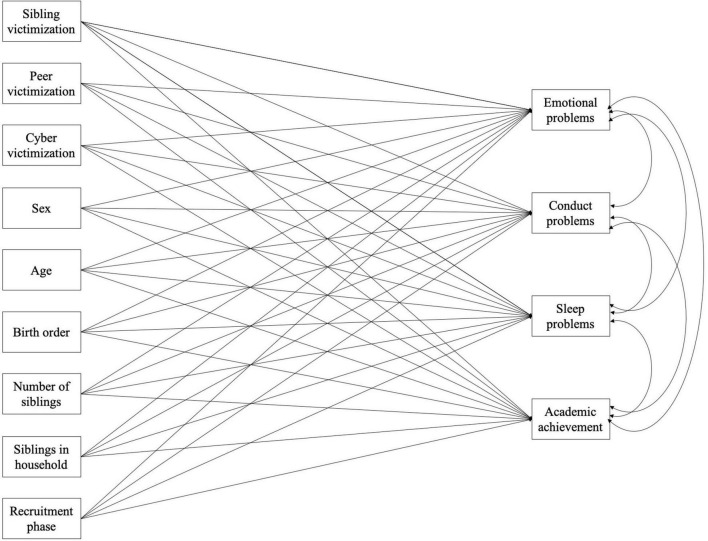
Conceptual depiction of the multivariate multiple regression model (MMR) as computed for Model 1. MMR allows for modelling the relationship between more than one independent variable (i.e., sibling, peer, and cyber victimization as well as control variables) and more than one outcome variable (i.e., emotional problems, conduct problems, sleep problems, and academic achievement) at the same time, thereby taking measurement error into account and guarding against type I error. Covariances among construct residuals of predictor variables were included in the model, but are not depicted for clarity.

IBM SPSS Statistics (IBM Corp, 2020) was utilized for all preliminary and descriptive analyses, while Mplus 8.1 (Muthén and Muthén, 2017) was used for all structural equation modeling (SEM). First descriptive statistics were utilized in order to compute frequencies of isolated and co-occurring forms of sibling, peer, and cyber bullying victimization and perpetration in the total sample and across gender (research question 1). Next, bivariate correlations were calculated across sibling, peer, and cyber bullying (victimization and perpetration) contexts in order to test the associations between the different bullying forms (research question 2). Finally, in order to explore independent (research question 3) and cumulative (research question 4) associations between bullying and child adjustment, SEM was employed (see Figure 1 for Model 1 as an example). Specifically, multivariate multiple regression (MMR) models were computed. MMR allows for modeling the relationship between more than one independent variable (i.e., sibling, peer, and cyber bullying as well as control variables) and more than one outcome variable (i.e., emotional problems, conduct problems, sleep problems, and academic achievement) at the same time. MMR is a particularly powerful multivariate methodology as it can take measurement errors into account and likewise guards against type I error. In order to assess whether sibling, peer, and cyber bullying were independently associated with child adjustment (research question 3), two separate MMR models were fitted, one for victimization (Model 1) and another for perpetration (Model 2). Sibling, peer, and cyber bullying were entered simultaneously into each model in order to control for one another. Sex, age, birth order, number of siblings, whether siblings lived in the same household, and recruitment phase were included as control variables. All four adjustment outcomes were entered together as outcome variables. Finally, in order to test whether there is a cumulative relationship between bullying victimization and perpetration across the sibling, peer, and cyber context and child adjustment (research question 4) a third MMR model was fitted (Model 3). For this purpose, a victimization index and a perpetration index were computed in order to reflect cumulative bullying across contexts (with each unit increase corresponding to an additional bullying context). The victimization and perpetration index were entered together as independent variables in addition to all control variables. The four adjustment outcomes were entered as outcome variables. Collinearity diagnostics were performed using the “collin” command in Stata (StataCorp, 2015). In order to check for multicollinearity, the variance inflation factor (VIF) across all independent variables was computed. All values indicated that there was no significant multicollinearity according to the criterion of VIF ≥ 10 by O’brien (2007). Further details can be found in the Supplementary Table 3.

## Results

### Research Question 1: Frequencies of Isolated and Co-occurring Forms of Sibling, Peer, and Cyber Bullying

**FIGURE 2:**
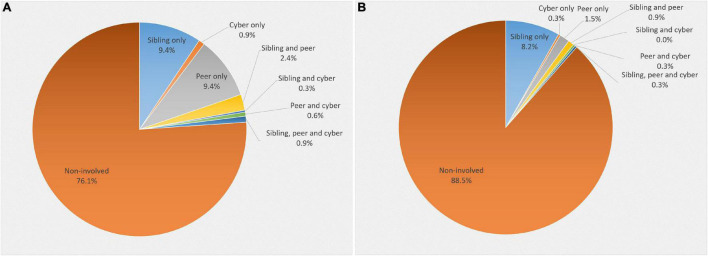
Prevalence of isolated and co-occurring forms of sibling, peer, and cyber bullying (*N* = 329). **(Chart A)** Bullying victimization across contexts. **(Chart B)** Bullying perpetration across contexts.

Of the total sample, 24% of children and adolescents reported experiencing frequent bullying victimization (more than three times in the past 6 months) in at least one context, while 11.6% of youth reported perpetrating frequent bullying in at least one context. The frequencies of youth involved in sibling, peer, and cyber bullying in the total sample and across gender are summarized in Table 1. Peer and sibling victimization were reported about equally, while cyber victimization was reported least often. Bullying perpetration, on the other hand, was most frequent in the sibling context, followed by the peer and cyber context. Chi-square tests revealed no significant gender differences across bullying contexts. A graphic illustration of the frequencies of isolated and co-occurring forms of sibling, peer, and cyber victimization and perpetration, respectively, is depicted in Figure 2. Isolated forms of sibling and peer victimization, as well as isolated forms of sibling perpetration were found to be the most frequently reported bullying forms. Only three children (0.9%) reported victimization in the sibling, peer, and cyber context simultaneously, and only one child (0.3%) reported perpetration in all three contexts.

**TABLE 1:** Prevalence of children and adolescents involved in sibling, peer, and cyber bullying in the total sample and across gender (*N* = 329).

	Total sample	Girls	Boys
Victimization			
Sibling	43 (13.1)	23 (14.2)	20 (12.0)
Peer	44 (13.4)	19 (11.7)	25 (15.1)
Cyber	9 (2.7)	5 (3.1)	3 (1.8)
Perpetration			
Sibling	31 (9.4)	15 (9.3)	16 (9.6)
Peer	10 (3.0)	4 (2.5)	6 (3.6)
Cyber	3 (.9)	0 (0)	3 (1.8)

*Results reported as n (%).*

*There was no missing data across bullying variables.*

*Bullying was coded as present if reported more than three times in the past 6 months.*

*Bullying was coded separately across contexts.*

*Thus, each child can be involved in more than one form of bullying.*

### Research Question 2: Associations Between Sibling, Peer, and Cyber Bullying

Bivariate correlations revealed a homotypic relationship between bullying victimization across contexts: Sibling, peer, and cyber victimization were all found to be intercorrelated (see Table 2). In other words, the more youth experienced victimization in one context, the higher was the likelihood that they were also victimized in another context. A homotypic relationship between bullying perpetration was found only between the sibling and peer context on the one hand, and between the peer and cyber context on the other hand. Further, bullying victimization and perpetration were found to be correlated within the sibling and cyber context. These correlations were particularly high within the sibling context, suggesting that youth involved in sibling bullying appeared to display particularly high concurrent victimization and perpetration behavior. Last, sibling victimization and peer perpetration were correlated, indicating that the more youth are victimized by their siblings, the more they perpetrated bullying in the peer context.

**TABLE 2:** Correlations across sibling, peer, and cyber bullying victimization and perpetration (*N* = 329).

	Bivariate correlations				
	1	2	3	4	5
1. Sibling victimization	-				
2. Peer victimization	0.14*	-			
3. Cyber victimization	0.16**	0.21***	-		
4. Sibling perpetration	0.62***	0.09	−0.05	-	
5. Peer perpetration	0.14*	0.09	0.08	0.19**	-
6. Cyber perpetration	0.06	0.06	0.38***	0.08	0.36***

*p < 0.05, **p < 0.01, ***p < 0.001 (two-tailed).

*There was no missing data across bullying variables.*

*Bullying was coded as present if reported more than three times in the past 6 months.*

*Bullying was coded separately across contexts.*

*Thus, each child can be involved in more than one form of bullying.*

### Research Question 3: Associations Between Sibling, Peer, and Cyber Bullying and Child Outcomes

Descriptive statistics of child outcomes across different contexts of bullying victimization and perpetration for the total sample are shown in the Supplementary Tables 1, 2. Results from the MMR models revealed that sibling and peer victimization were associated with more emotional problems, conducts problems, and sleep problems, while cyber victimization was linked to more conduct problems and higher academic achievement, even after accounting for sex, age, birth order, number of siblings, whether siblings lived in the same household, and recruitment phase (see Table 3; Model 1). Overall, 19,8% of variance for emotional problems, 19.2% for conduct problems, 9.1% for sleep problems, and 9.5% for academic achievement could be explained. Findings in relation to bullying perpetration indicated that peer perpetration was related to more emotional and sleep problems, while cyber perpetration was associated with more conduct problems and better academic achievement, even after controlling for confounders (see Table 3; Model 2). Sibling perpetration was not associated with any of the child outcomes. Taken together, 10.9% of variance for emotional problems, 9.4% for conduct problems, 4.3% for sleep problems, and 8.6% for academic achievement could be explained. Concerning the control variables, it was found that males were found to display lower levels of emotional and sleep problems, higher levels of conduct problems, and better academic achievement compared to females. Moreover, the older the youth, the more emotional problems they reported. Children with more siblings were further found to report better academic achievement. The recruitment phase was only found to be associated with academic achievement, with children and adolescents recruited during the second phase reporting lower levels of academic achievement. Associations between control variables and outcome variables were similar across models 1 and 2.

**TABLE 3:** Multivariate multiple regression analysis of the independent and cumulative associations between bullying and child outcomes (*N* = 329).

	Outcome variables															

	Emotional problems				Conduct problems				Sleep problems				Academic achievement			
Model 1	*B*	SE	β	*p*	*B*	SE	β	*p*	*B*	SE	β	*p*	*B*	SE	β	*p*
Sex	−0.23	0.06	−0.25	**<0.001**	0.10	0.05	0.14	**0.029**	−0.16	0.07	−0.16	**0.023**	−0.36	0.11	−0.23	**0.001**
Age	0.05	0.02	0.14	**0.006**	0.02	0.01	0.06	0.238	−0.01	0.02	−0.01	0.794	−0.06	0.04	−0.10	0.088
Birth order	−0.05	0.05	−0.05	0.330	0.02	0.04	0.03	0.616	−0.07	0.06	−0.06	0.251	−0.09	0.09	−0.06	0.318
Number of siblings	0.05	0.03	0.09	0.084	0.01	0.02	0.02	0.647	−0.03	0.03	−0.05	0.331	−0.14	0.05	−0.16	**0.006**
Siblings in household	−0.04	0.07	−0.03	0.559	0.01	0.05	0.02	0.781	−0.10	0.08	−0.07	0.233	0.02	0.13	0.01	0.895
Recruitment phase	0.03	0.07	0.03	0.631	0.03	0.05	0.04	0.564	0.10	0.08	0.09	0.191	0.30	0.13	0.17	**0.016**
Sibling victimization	0.18	0.07	0.13	**0.014**	0.15	0.05	0.15	**0.005**	0.19	0.08	0.12	**0.023**	−0.16	0.13	−0.07	0.222
Peer victimization	0.41	0.07	0.29	**<0.001**	0.22	0.05	0.22	**<0.001**	0.30	0.08	0.20	**<0.001**	0.09	0.13	0.04	0.496
Cyber victimization	0.05	0.16	0.02	0.780	0.49	0.12	0.23	**<0.001**	0.20	0.18	0.06	0.273	−0.74	0.28	−0.15	**0.009**
**Model 2**																
Sex	−0.22	0.07	−0.23	**0.001**	0.11	0.05	0.15	**0.027**	−0.14	0.07	−0.15	**0.042**	−0.34	0.47	−0.22	**0.003**
Age	0.05	0.02	0.13	**0.019**	0.01	0.02	0.04	0.527	−0.01	0.02	−0.03	0.663	−0.05	0.04	−0.08	0.153
Birth order	−0.04	0.05	−0.04	0.455	0.03	0.04	0.05	0.397	−0.06	0.06	−0.05	0.339	−0.10	0.09	−0.06	0.261
Number of siblings	0.04	0.03	0.08	0.138	0.01	0.02	0.04	0.523	−0.03	0.03	−0.06	0.321	−0.15	0.05	−0.16	**0.005**
Siblings in household	−0.06	0.07	−0.04	0.450	0.02	0.05	0.02	0.742	−0.11	0.08	−0.08	0.177	0.01	0.13	0.00	0.943
Recruitment phase	0.01	0.07	0.01	0.889	0.00	0.05	0.00	0.952	0.08	0.08	0.07	0.332	0.33	0.12	0.19	**0.008**
Sibling perpetration	0.11	0.09	0.07	0.230	0.11	0.64	0.10	0.079	0.15	0.10	0.09	0.128	−0.04	0.15	−0.01	0.813
Peer perpetration	0.41	0.16	0.15	**0.009**	0.16	0.12	0.08	0.164	0.36	0.18	0.12	**0.039**	0.00	0.26	0.00	0.997
Cyber perpetration	−0.32	0.28	−0.06	0.267	0.63	0.21	0.17	**0.002**	−0.11	0.31	−0.02	0.728	−1.23	0.47	−0.15	**0.010**
**Model 3**																
Sex	−0.23	0.06	−0.23	**<0.001**	0.11	0.05	0.15	**0.019**	−0.16	0.07	−0.15	**0.024**	−0.36	0.11	−0.22	**0.001**
Age	0.05	0.02	0.13	**0.009**	0.02	0.01	0.06	0.235	−0.01	0.02	−0.02	0.722	−0.06	0.03	−0.10	0.077
Birth order	−0.05	0.05	−0.05	0.343	0.02	0.04	0.02	0.569	−0.06	0.06	−0.06	0.270	−0.09	0.09	−0.05	0.322
Number of siblings	0.05	0.03	0.09	0.118	0.02	0.02	0.04	0.449	−0.03	0.03	−0.05	0.303	−0.15	0.05	−0.16	**0.003**
Siblings in household	−0.04	0.07	−0.02	0.621	0.02	0.05	0.01	0.765	−0.09	0.08	−0.06	0.236	0.02	0.13	0.01	0.872
Recruitment phase	0.04	0.07	0.03	0.578	0.02	0.05	0.04	0.743	0.10	0.08	0.08	0.191	0.33	0.13	0.17	**0.009**
Victimization index	0.21	0.04	0.31	**<0.001**	0.15	0.03	0.29	**<0.001**	0.18	0.04	0.25	**<0.001**	−0.06	0.07	−0.04	0.393
Perpetration index	0.01	0.06	0.01	0.885	0.09	0.04	0.12	**0.033**	0.07	0.07	0.06	0.299	−0.12	0.10	−0.08	0.229

*Significant values are in bold (p < 0.05).*

*B, Unstandardized estimates; SE, Standard error; β, Standardized coefficients.*

*Full information maximum likelihood (FIML) was applied.*

*Sex: 0 = female, 1 = male; Age (in years); Birth order: 0 = later born, 1 = first born; Number of siblings (counting numbers); Siblings in household: 0 = no, 1 = yes; Recruitment phase: 0 = recruitment phase 1, 1 = recruitment phase 2.*

*Children’s involvement in bullying victimization and perpetration within each context was coded as present if reported more than three times in the past 6 months.*

*The victimization index (0–3) reflects cumulative victimization (with each unit increase corresponding to an additional bullying form).*

*The perpetration index (0–3) reflects cumulative perpetration (with each unit increase corresponding to an additional bullying form).*

### Research Question 4: Cumulative Associations Between Bullying Victimization and Perpetration Across Contexts and Child Outcomes

The final MMR model illustrating the cumulative relationship between bullying victimization and perpetration (with each unit increase corresponding to an additional bullying form) across the sibling, peer, and cyber context and child outcomes is summarized in Table 3 (Model 3). Results suggest that cumulative bullying victimization was associated with higher levels of emotional problems, conduct problems, and sleep problems, indicating that youth who experienced multiple forms of bullying victimization were at the greatest risk of poor adjustment, even after controlling for confounders. Findings in relation to cumulative bullying perpetration indicated that perpetration across multiple bullying contexts was linked to more conduct problems, after considering the controls. Control variables were associated with child outcomes in the comparable strength and direction as described above. Overall, when considering cumulative forms of bullying victimization and perpetration as well as all control variables, 18,5% of variance for emotional problems, 15,5% for conduct problems, 9,3% for sleep problems, and 7,6% for academic achievement could be explained.

## Discussion

The present study is one of the first to provide an integrated overview of the frequencies of isolated and co-occurring forms of bullying victimization and perpetration across the sibling, peer, and cyber context among children and adolescents. Associations between bullying in the home, bullying in school, and bullying online were examined. Moreover, comparative associations between independent and cumulative associations of sibling, peer, and cyber bullying with emotional problems, conduct problems, sleep problems, and academic achievement were investigated in order to establish whether different bullying forms contributed uniquely to child adjustment and whether a dose-response relationship could be identified.

### Frequencies Across Bullying Contexts

Our findings in relation to the frequencies of bullying across contexts were partially in line with our hypothesis. We found that around 13% of children and adolescents reported experiencing frequent sibling and peer victimization, while only around 3% reported cyber victimization. Thus, against our expectation sibling and peer victimization were reported equally frequent, whereas past research has shown that victimization in the sibling context occurs more frequently than in the peer context (Finkelhor et al., 2015). The proportions of youth reporting bullying perpetration, however, were in accordance with our expectations: Sibling perpetration was reported most frequently; by about one out of ten children and adolescents in our sample, while peer perpetration was reported second most frequently by 3% of youth, while cyber perpetration was reported only by around 1%. The occurrence of bullying across contexts in our sample is comparatively low in the light of previous studies (Modecki et al., 2014; Wolke et al., 2015; Jadambaa et al., 2019). Why might the occurrence of bullying in our sample be lower than previously reported? One of the greatest challenges of prevalence studies on bullying is the lack of a uniform definition as well as a standardized instrument that assess bullying with the same time reference period and frequency criterion (Wolke et al., 2015; Menesini and Salmivalli, 2017). This leads to a wide variety of prevalence estimates, making it difficult to compare results across studies. Another reason for the inconsistency may stem from our use of a single-item scale, which could have biased reports. However, regarding sibling bullying previous studies have found that single-item scales and multi-item scales are highly correlated (Dantchev et al., 2018; Toseeb et al., 2020). While the validity of the single-item approach has been confirmed (Catone et al., 2019), authors have still made recommendations to use validated multiple-item scales, in addition to single items, in order to make more precise estimates and cross-national comparisons (Yanagida et al., 2016). It may, however, also be that the frequency of bullying involvement was generally lower in the current sample of Austrian youth. That said, one must keep in mind that we cannot claim that our findings are based on representative data. Moreover, it is important to consider that data collection took place during the COVID-19 pandemic and thus frequencies may be inaccurate. Scholars have argued that the risk for engaging in sibling aggression in the time of COVID-19 has been particularly exacerbated (Perkins et al., 2021). Contrary to this, data assessing peer and cyber bullying prevalence rates during the pandemic has shown that involvement in all forms of bullying were reported at far lower rates during the pandemic compared to before the pandemic, except for cyber bullying (Vaillancourt et al., 2021).

We had further expected that youth would report co-occurring bullying more frequently compared to isolated bullying forms. Contrary to this, we found that isolated sibling and peer victimization were reported most frequently in our sample, followed by isolated sibling perpetration. Surprisingly, under 1% of children and adolescents reported co-occurring sibling, peer, and cyber bullying. The present study nevertheless adds to the literature by underlining the ubiquity of sibling bullying involvement and calls for equal attention to be assigned to this specific form of aggression, as it has been given to peer and cyber bullying.

### Associations Across Bullying Contexts

We found that bullying victimization and perpetration were largely associated in a homotypic manner across contexts. In other words, youth who were victimized at the hands of their siblings were also more often victimized by their peers and in the cyber context. Similarly, youth who bullied their peers at school also reported perpetrating more bullying online and at home. These findings resonate with previous work that have also found a homotypic relationship between sibling and peer bullying (Tippett and Wolke, 2015; Dantchev et al., 2019; Tucker et al., 2019). They are also consistent with previous work showing that peer and cyber victimization, but also peer and cyber perpetration were associated (Modecki et al., 2014; Walters, 2021). The present study adds to the current literature by investigating the relationship between sibling and cyber bullying for the first time, showing that sibling victimization is also linked to cyber victimization. We further found that victimization and perpetration were highly correlated within the sibling and cyber context suggesting that many youths were involved in both roles at the same time (i.e., bully-victims).

### Associations Between Bullying and Child Adjustment

#### Emotional Problems

There is considerable evidence indicating that children and adolescents who are affected by bullying are at heightened risk of internalizing problems (Reijntjes et al., 2010; Moore et al., 2017). Our study found that sibling victimization and any involvement in peer bullying (i.e., victimization and perpetration) were found to be associated with more emotional problems, mirroring previous work. Cyber bullying involvement on the other hand, was not found to be linked with emotional problems. A meta-analysis comparing the prospective links between peer and cyber bullying with internalizing problems found that that peer and cyber victimization contributed uniquely to increased levels of internalizing symptoms, even after controlling for one another (Gini et al., 2018). These findings contradict ours. It is possible that our single-item scale was not sensitive enough to capture the effects for cyber victimization. Findings in relation to our control variables may likewise provide possible explanations for our discrepant findings and inform future work at the same time. We found that females and older youth reported higher levels of emotional problems compared to males and younger youth. Hence, it may be important for future work to replicate our findings and consider including gender and age as moderators in the relationship between bullying across contexts and emotional problems. Consistent with previous work, we further identified a cumulative association between bullying victimization and emotional problems (Tucker et al., 2014; Wolke et al., 2017; Dantchev et al., 2019; Sharpe et al., 2021). Our findings thus suggest that while sibling and peer victimization appear to have independent contributions toward experiencing emotional problems, cyber victimization may need to co-occur with other forms of bullying in order to have an influence. Finally, above all, our results highlight the importance of investigating multiple bullying forms across contexts simultaneously. Mapping onto previous work, we too show that bullying as a trauma (Idsoe et al., 2021; McKay et al., 2021), may be most harmful when experiences across multiple contexts, as this means that young people have no safe place to escape (Dantchev et al., 2019).

#### Conduct Problems

The current study found that youth who reported acting as the aggressor in the cyber context were found to show heightened levels of conduct problems. However, sibling and peer bullying perpetrating were not found to be independently linked to conduct problems. This is surprising, as there are a number of studies reporting an association between sibling and peer bullying perpetration and externalizing problems (Wolke and Samara, 2004; Toseeb et al., 2020). Interestingly, our results further indicated that involvement in any bullying victimization (sibling, peer, and cyber context) was uniquely associated with more conduct problems. This may seem counterintuitive and contradictory in the light of past research. However, the bullying literature has suggested that rather than bullying victimization *per se* being the leading risk factor for externalizing problems, it may be that the specific combination of becoming victimized and fighting back (i.e., the sub-group of bully-victims) that are driving the links (Wolke et al., 2013; Foody et al., 2020). However, the majority of studies have not included this specific sub-group of children and adolescents (Klomek et al., 2015) and thus the reasons remain speculative.

The findings further suggested that both cumulative bullying victimization and perpetration across contexts revealed a dose-response relationship with conduct problems. Hence, while sibling and peer bullying alone did not contribute to conduct problems, acting as an aggressor across multiple contexts was linked to poorer outcomes. These findings are line with previous studies that have reported that youth that perpetrate both sibling and peer bullying are at a greater risk of developing externalizing problems (Foody et al., 2020) and high-risk behavior including criminal behavior and illicit drug use (Dantchev and Wolke, 2019). Other studies on the other hand have reported that children and adolescents that are victimized concurrently by peers and in the cyber context have been reported to display the greatest behavioral problems (Wolke et al., 2017). In line with general strain theory (Agnew, 1992), bullying victimization has been proposed as a significant strain which may result in delinquent behavior (Barbieri et al., 2019). Thus, as indicated through our findings, victimization across the sibling, peer, and cyber context may exacerbate the experienced strain, thereby leading to more conduct problems. Social learning theory (Bandura, 1977) can further inform ways in which cumulative bullying perpetration can result in heightened conduct problems. In line with social learning theory behavior that is positively reinforced will be internalized as adaptive and modeled across other contexts. Taken together, findings in relation to conduct problems highlight the predicament of youth who are found to experience bullying victimization and perpetration consistently across multiple social contexts.

#### Sleep Problems

There is convincing evidence that peer victimization is a risk factor for increased levels of sleep problems during childhood and adolescence (van Geel et al., 2016; Zimmer-Gembeck et al., 2019). Findings in relation to peer perpetration (Na and Park, 2018; Hysing et al., 2021) as well as cyber bullying involvement (Jose and Vierling, 2018), however, are scarce and less clear. In line with our hypothesis, we found that sibling and peer victimization were independently associated with more sleep problems. Similarly, peer perpetration was linked to increased levels of sleep problems. However, contrary to what we expected, cyber victimization was not found to be uniquely associated with sleep problems. Although some previous work has found evidence that cyber victimization is linked with sleep problems in childhood (Jose and Vierling, 2018) and adolescence (Donoghue and Meltzer, 2018), other forms of bullying were not accounted for in these studies (Kowalski et al., 2014). Moreover, we could identify a cumulative relationship between youth reporting multiple forms of bullying victimization, but not bullying perpetration, and more sleep problems in our sample. Thus, results from our final model in which cumulative bullying victimization and perpetration have been accounted for simultaneously suggest that victimization experiences are particularly pertinent for sleep problems. There has been previous longitudinal work showing that a greater increase in peer victimization over time is associated with increasing sleep problems over time (Chang et al., 2018). Our results add to this and suggest that similar dose-response associations may exist for experiencing multiple forms of victimization, even after accounting for perpetration. However, we examined sleep problems as one of four child outcomes but did not consider it as a potential mediator. While bullying experiences may directly influence sleep quality *via* mechanisms of rumination, worry and distress for instance (van Geel et al., 2016; Jose and Vierling, 2018), studies have specifically found sleep quality to mediate the link between bullying and mental health (Tu et al., 2015; Tu et al., 2019), externalizing behavior (Sosnowski et al., 2016), as well as academic achievement (Hysing et al., 2021). Sleep should thus be considered as a possible mechanism that can help explain the ways in which bullying may result in poor child adjustment.

#### Academic Achievement

While there appears to be consistent evidence about the negative association between peer victimization and academic achievement (Nakamoto and Schwartz, 2010; Moore et al., 2017), there is far less work on peer perpetration as well as cyber and sibling bullying in relation to academic achievement. Contrary to our hypothesis, we did not find an independent or cumulative association between sibling or peer bullying victimization and academic achievement. While there is some evidence that resemble our findings (Evans et al., 2018), they are largely contrary to what was expected on the basis of a bulk of previous work on peer bullying (Nakamoto and Schwartz, 2010). Similarly, against our expectations we found that cyber victimization and cyber perpetration were associated with better academic achievement. Some scholars have speculated that victimized youth may engage more academically in order to compensate or to find positive status elsewhere (Evans et al., 2018). In regard to cyber perpetration, online engagement has been identified as a key predictor of cyber perpetration (Kowalski et al., 2014). Considering the increased online presence associated with the COVID-19 pandemic, it is plausible that youth in our sample were naturally more susceptible to engage in more cyber perpetration. Along these lines, it is important to consider that the data in this study was collected during the pandemic and the majority of youth were affected by alternating periods of home schooling. School closures were incurred around mid-March 2020 in Austria and replaced by a hybrid solution around mid-May 2020. Hybrid solutions involved alternating periods during which half of the class was permitted to attend school while the other half attended digital home schooling sessions. As of February 2021, another period of hybrid schooling was enforced. In order to account for these schooling irregularities, we controlled for the corresponding recruitment phase (Phase 1: April–June 2020; Phase 2: January–April 2021). Youth recruited during the second phase were indeed found to report lower levels of academic achievement overall. There is evidence showing that youth have experienced a learning loss during lockdown (Engzell et al., 2021). It is thus possible that children and adolescents who were recruited during the second phase with a sustained history of home schooling suffered greater academic challenges. The current results, particularly concerning bullying in the cyber context and academic achievement, must therefore be interpreted with caution. With participants experiencing home schooling to a various degree and by contingency increased use of electronic media, it is feasible that the significant association between cyber victimization and perpetration and better academic achievement is not generalizable to non-pandemic times.

### Practical Implications

The current study has several practical implications. Our findings on the associations between bullying forms and contexts call for early detection of bullying in the home as a promising route toward reducing and preventing spillover onto other contexts. Longitudinal data support this notion, with scholars reporting that sibling victimization in early childhood may precede later peer victimization (Tucker et al., 2019). Furthermore, the current study contributes to the literature by underlining the significant and unique contributions that sibling bullying may have, over and above peer and cyber bullying. Sibling bullying is only beginning to emerge as an additionally accepted childhood trauma that may influence the development of mental health problems prospectively (McKay et al., 2021), thus there is an urgent need for future prospective studies on bullying that include the sibling context routinely. While peer and cyber bullying are placed more firmly on the research agenda, sibling bullying continues to be greatly neglected in the domain of anti-bullying programs. Our findings strongly support the need for novel approaches in dealing with bullying in order to prevent future developmental trajectories of mental health problems and antisocial behavior (Kretschmer et al., 2014; Le et al., 2018; Haahr-Pedersen et al., 2020). While there is a handful of interventions that have been developed to reduce sibling conflict, there are currently no interventions that have been tailored toward reducing sibling bullying specifically (Tucker and Finkelhor, 2017). Targeting intervention work tailored toward sibling relationships and bullying early on within the home may hold promising outlooks on reducing and preventing peer and cyber bullying as well. We argue toward adopting multi-modal approaches that include training and interventions components targeting sibling, peer, and cyber bullying separately and simultaneously in efforts to prevent and reduce bullying globally. Whether and how such interventions can be best implemented relies on future studies that continue to investigate these three bullying forms simultaneously.

### Limitations

This study has some limitations that merit consideration. The cross-sectional design remits caution when interpreting the results, as causal inferences cannot be made. Moreover, the study sample was relatively small precluding representative prevalence estimates. It is important to conduct larger and nationally representative population studies that assess all three bullying contexts in order to gain a deeper understanding for the prevalence of and associations between the multiple bullying forms. Importantly, the study sample did not permit a thorough exploration of the different bullying roles (non-involved, victim, bully, bully-victims) as a result of the limited sample size. It is imperative to better explore the differential links to child outcomes separately for each bullying role across the three contexts in the future, as there is a bulk of evidence showing that bullying is differentially associated with child adjustment depending on the role, context, and outcomes examined (Haynie et al., 2001; Wolke et al., 2013; Wolke and Lereya, 2015). Our study investigated outcomes in relation to victimization and perpetration separately, therefore it is possible that specific links attributable to bully-victims were disguised (Kowalski et al., 2014). Moreover, all measures employed in the current study were based on self-report only, therefore a common-method bias cannot be excluded. However, the literature shows that sibling, peer, and cyber bullying typically occur behind closed doors (Holt et al., 2008; Demaray et al., 2013; Byrne et al., 2014; Wolke et al., 2015) and parents are often not entirely aware of the problem. Future work comparing child and parental reports of bullying across these three contexts is necessary to shed more light on this matter. Regarding our measures, it should be noted that the internal consistency of the conduct problem subscale was comparatively low. While this appears to be a common pattern amongst empirical work using this subscale across childhood (Van Roy et al., 2008), caution regarding the interpretation of results is warranted. Last, it is important to consider that the COVID-19 pandemic as well as the alternating periods of home schooling may have influenced the findings of this study. The pandemic has been associated with greater child and adolescent mental health problems and learning loss as compared to pre-pandemic data (Engzell et al., 2021; Ravens-Sieberer et al., 2021), especially for youth who are disproportionally burdened by the circumstance such as those growing up in disadvantaged homes. Although our study accounted for the recruitment phase of youth, we were unable to consider socioeconomic status or specific schooling contexts of individuals, thus we cannot exclude these contextual factors as possible confounders that may have influenced our findings.

## Conclusion

Sibling, peer, and cyber bullying in childhood and adolescence are found to highly co-occur. Moreover, bullying at home, in school, and online were uniquely and cumulatively associated with poor adjustment. Bullying across all three contexts must therefore be integrated and placed more firmly on the research agenda. Psychoeducation needs to be provided for parents, teachers, and health professionals in order to sensitize them toward sibling bullying and help them identify this problematic behavior. This is important as supporting early identification of bullying in the home may hold great promise toward reducing and preventing bullying to spill over into the school and the cyber context. Last, multi-modal anti-bullying prevention and intervention programs, which are tailored to bullying across these three contexts, are urgently needed in order to appropriately target bullying behavior across development and provide young people with tools to interact in healthy ways as well as respond toward aggressive behavior adaptively.

## Data Availability Statement

The datasets presented in this study can be found in online repositories. The names of the repository/repositories and accession number(s) can be found below: https://osf.io/8psrj/.

## Ethics Statement

The studies involving human participants were reviewed and approved by the Institutional Review Board of the University of Vienna (Reference Nr.: 00480; Date of approval: 19.11.2020). Written informed consent to participate in this study was provided by the participants’ legal guardian/next of kin.

## Author Contributions

SD drafted the manuscript and was responsible for the collection and analysis of the data. MZ provided critical reviews and revised the work. Both authors contributed to the design of the study and interpretation of the data.

## Conflict of Interest

The authors declare that the research was conducted in the absence of any commercial or financial relationships that could be construed as a potential conflict of interest.

## Publisher’s Note

All claims expressed in this article are solely those of the authors and do not necessarily represent those of their affiliated organizations, or those of the publisher, the editors and the reviewers. Any product that may be evaluated in this article, or claim that may be made by its manufacturer, is not guaranteed or endorsed by the publisher.
