# Supplementary material for: Sibling, Peer, and Cyber Bullying Among Children and Adolescents: Co-occurrence and Implications for Their Adjustment
Source: Front Psychol. 2021 Dec 22;12:761276. doi: 10.3389/fpsyg.2021.761276 (PMC8728088; doi:10.3389/fpsyg.2021.761276)
Supplement: Supplementary file 1 [file Table_1.DOCX]

Supplemental Material

| Table S1. | | | | | | |  |
| --- | --- | --- | --- | --- | --- | --- | --- |
| *Descriptive statistics of child outcomes across different contexts of bullying victimization involvement (N = 329)* | | | | | | |  |
|  | Sibling context | | Peer context | | Cyber context | | |
|  | Non-involved | Involved | Non-involved | Involved | Non-involved | Involved |  |
| Emotional problems | 1.53 (.47) | 1.76 (.52) | 1.51 (.46) | 1.88 (.55) | 1.55 (.48) | 1.84 (.61) |  |
| Conduct problems | 1.37 (.32) | 1.54 (.38) | 1.35 (.30) | 1.51 (.40) | 1.37 (.31) | 1.73 (.44) |  |
| Sleep problems | 1.48 (.50) | 1.73 (.63) | 1.47 (.48) | 1.69 (.59) | 1.49 (.50) | 1.70 (.74) |  |
| Academic achievement | 3.83 (.77) | 3.67 (.85) | 3.87 (.76) | 3.70 (.94) | 3.86 (.78) | 3.46 (1.07) |  |
| *Note:* Results reported as *M (SD).* Children’s involvement with bullying victimization within each context was coded as present if reported more than three times in the past six months. | | | | | | |  |

| Table S2. | | | | | | |  |
| --- | --- | --- | --- | --- | --- | --- | --- |
| *Descriptive statistics of child outcomes across different contexts of bullying perpetration involvement (N = 329)* | | | | | | |  |
|  | Sibling context | | Peer context | | Cyber context | | |
|  | Non-involved | Involved | Non-involved | Involved | Non-involved | Involved |  |
| Emotional problems | 1.54 (.46) | 1.74 (.57) | 1.55 (.48) | 1.93 (.49) | 1.56 (.49) | 1.52 (.27) |  |
| Conduct problems | 1.38 (.33) | 1.49 (.34) | 1.36 (.31) | 1.73 (.45) | 1.37 (.31) | 2.00 (.49) |  |
| Sleep problems | 1.49 (.50) | 1.70 (.63) | 1.49 (.50) | 1.76 (.44) | 1.50 (.50) | 1.40 (.57) |  |
| Academic achievement | 3.82 (.78) | 3.75 (.84) | 3.86 (.78) | 3.52 (.88) | 3.86 (.78) | 2.90 (.63) |  |
| *Note.* Results reported as *M (SD).* Children’s involvement with bullying perpetration within each context was coded as present if reported more than three times in the past six months. | | | | | | |  |

| Table S3. | | | |
| --- | --- | --- | --- |
| *Variance inflation factors across all predictors for all models (N = 329)* | | | |
|  | VIF | | |
|  | Model 1 | Model 2 | Model 3 |
| Sex | 1.72 | 1.72 | 1.71 |
| Age | 1.04 | 1.05 | 1.02 |
| Birth order | 1.08 | 1.08 | 1.08 |
| Number of siblings | 1.10 | 1.10 | 1.09 |
| Siblings in household | 1.06 | 1.07 | 1.06 |
| Recruitment phase | 1.67 | 1.66 | 1.67 |
| Sibling victimization | 1.07 | - | - |
| Peer victimization | 1.07 | - | - |
| Cyber victimization | 1.12 | - | - |
| Sibling perpetration | - | 1.08 | - |
| Peer perpetration | - | 1.19 | - |
| Cyber perpetration | - | 1.17 | - |
| Victimization index | - | - | 1.11 |
| Perpetration index | - | - | 1.12 |

*Note.* VIF = Variance inflation factor. A VIF ≥ 10 indicates significant multicollinearity (O’Brien, 2007).


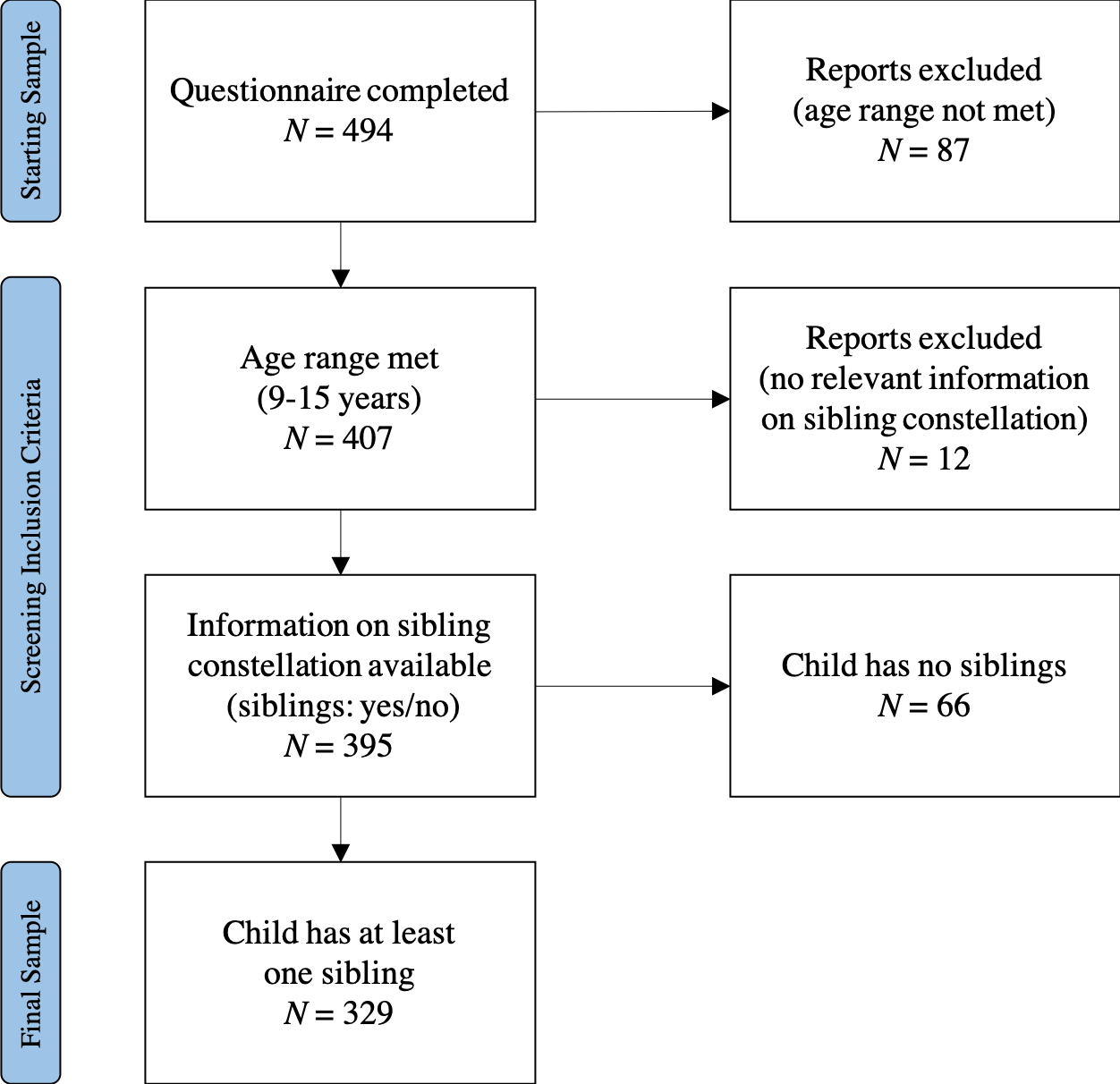


Figure S1. Participant flowchart illustrating the achieved study sample size.
